# Author Correction: Lifecycle of a predatory bacterium vampirizing its prey through the cell envelope and S-layer

**DOI:** 10.1038/s41467-025-58758-7

**Published:** 2025-04-15

**Authors:** Yoann G. Santin, Adrià Sogues, Yvann Bourigault, Han K. Remaut, Géraldine Laloux

**Affiliations:** 1 de Duve Institute, UCLouvain, 75 avenue Hippocrate, 1200 Brussels, Belgium; 2 Structural and Molecular Microbiology, Structural Biology Research Center, VIB, Pleinlaan 2, 1050 Brussels, Belgium; 3 Structural Biology Brussels, Vrije Universiteit Brussel, Pleinlaan 2, 1050 Brussels, Belgium

Correction to: *Nature Communications* 10.1038/s41467-024-48042-5, published online 27 April 2024

In the version of the article initially published, in the second paragraph, “including” was missing from the sentence now reading “…based on previous observations including that *Caulobacter crescentus* strains carrying an S-layer were not preyed upon”. In the first paragraph of the Discussion, the text “this statement lacked unambiguous evidence and systematic comparisons between isogenic prey strains. Based on the direct evidence presented in our study, we therefore recommend revisiting the role of the S-layer as a defensive barrier against predatory bacteria. Nonetheless,” has been removed. In the same paragraph, the sentence “we demonstrated that its prey range extends beyond *C. crescentus* but is confined to specific families within the α-proteobacterial class” has been amended to “we demonstrated that, within the α-proteobacterial class, its prey range extends beyond *C. crescentus* but is confined to specific families”. These corrections have been made to the HTML and PDF versions of the article.

