# Supplementary material for: Lifecycle of a predatory bacterium vampirizing its prey through the cell envelope and S-layer
Source: Nat Commun. 2024 Apr 27;15:3590. doi: 10.1038/s41467-024-48042-5 (PMC11055950; doi:10.1038/s41467-024-48042-5)
Supplement: Supplementary file 1 — Supplementary Information [file 41467_2024_48042_MOESM1_ESM.pdf]

SUPPLEMENTARY FIGURES

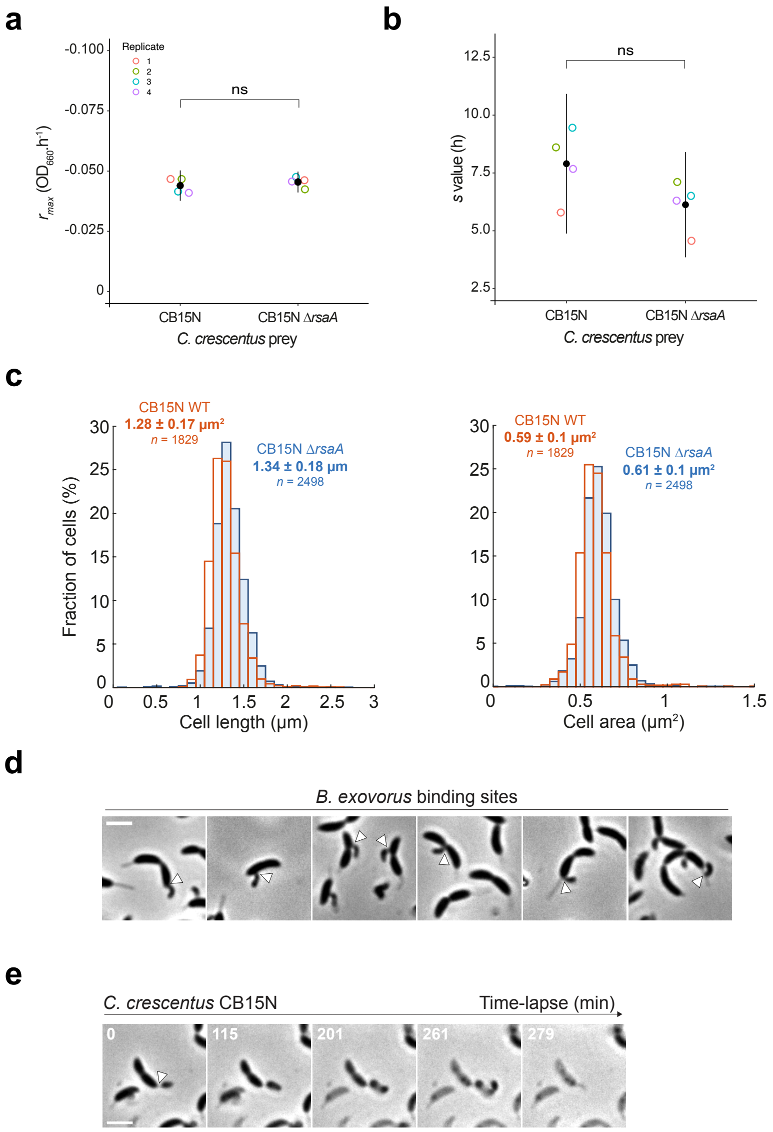

Supplementary Fig. 1

Supplementary Fig. 1 | The presence of the S-layer does not impact killing parameters, the predator binding site or the size of newborn predators. a-b, Dotplot representation of the *B. exovorus* r_{max} (**a**) and *s* (**b)** killing parameters in presence of the wild-type or the Δ*rsaA C. crescentus* strain as prey, obtained from replicates of the killing assay shown in Fig. 2b. The mean (filled black circle) and the standard deviation (black lines) are indicated. Pairwise comparison from four biological replicates is indicated above each plot (ns, nonsignificant; two-sample Fisher-Pitman permutation test). **c,** Histograms representing the indicated cellular dimensions of *B. exovorus* predators upon overnight predation of either the wild-type (CB15N WT, orange) or the Δ*rsaA C. crescentus* (CB15N Δ*rsaA*, blue) strain as a prey. Quantification based on the predator cells depicted in **Fig. 1a** and **Fig. 2a**. Values of the mean and the standard deviation, and the number of analyzed predator cells (*n*) are indicated on the graphs for each condition. Source data are provided as a Source Data file. **d**, Representative phase contrast images of *B. exovorus* cells attached to the wild-type *C. crescentus* upon 15 min of co-incubation. White arrowheads highlight predator binding sites. The last image on the right shows a predator cell on the *C. crescentus* stalk (see also Supplementary Movie 3 for a movie of the same cell). All selected binding sites result in predator growth. Scale bar, 2 µm. **e,** Representative time-lapse phase contrast microscopy images of a *B. exovorus* cell attached to, and growing on, the stalk of a wild-type *C. crescentus* cell (white arrowhead). Scale bar, 2 µm. Related to **Fig. 2**.

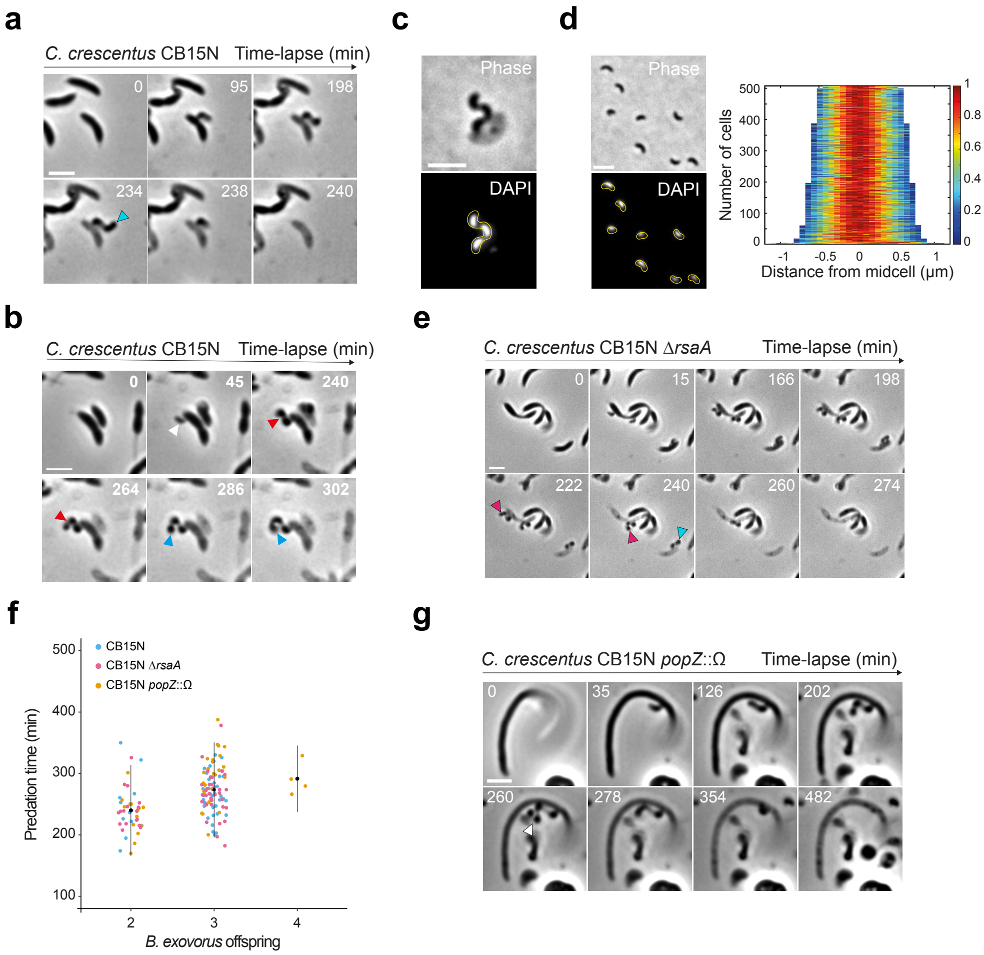

		CB15N	CB15N *ΔrsaA*	CB15N *popZ*::Ω
	2	245 ± 48 min	242 ± 31 min	232 ± 33 min
#	3	264 ± 34 min	266 ± 35 min	295 ± 42 min
	4	NA	NA	292 ± 27 min

Supplementary Fig. 2

Supplementary Fig. 2 | The predator offspring is unaffected by the presence of the S-layer or the prey cell size. a, Representative time-lapse phase contrast microscopy images of the *B. exovorus* grow th using the wild-type *C. crescentus* strain as a prey. The cyan arrowhead shows the production of 2 progenies. Scale bar, 2 µm. **b,** Representative time-lapse phase contrast microscopy images of *B. exovorus* growing onto a wild-type *C. crescentus* prey. The white arrowhead indicates attachment of a *B. exovorus* cell to a *C. crescentus* cell. Red arrows indicate the appearance of constriction sites. Blue arrowheads indicate predator growth after the formation of constriction sites. Scale bar, 2 µm. **c,** Representative microscopy image of a *B. exovorus* cell in the final predation stage on the surface of a ghost wild-type *C. crescentus* cell, showing future triplet progenies stained with DAPI. Top, phase contrast; bottom, DAPI fluorescence signal and *B. exovorus* cell outline drawn manually based on the phase contrast image. **d,** Left: Representative microscopy image of attack-phase *B. exovorus* upon overnight predation on wild-type *C. crescentus* and staining with DAPI. Top, phase contrast; bottom, DAPI fluorescence signal and cell outlines obtained with Oufti. Right: Demograph of the DAPI signal obtained from the same population of cells as on the left. The heatmap represents relative fluorescence intensities. Cells are sorted by length. **e**. Representative time-lapse phase contrast microscopy images of *B. exovorus* growth using the Δ*rsaA C. crescentus* strain as a prey. Cyan and magenta arrowheads show the production of 2 or 3 progenies, respectively. Scale bar, 2 µm. **f,** Dotplot representation of the *B. exovorus* predation time using either the wild-type (blue), Δ*rsaA* (pink), or *popZ*::Ω (orange) *C. crescentus* strain as a prey. Predation time is defined as the time interval between attachment of *B. exovorus* onto the prey and the escape of the last predator cell upon progeny release. Values of the mean (filled black circle) and the standard deviation (black lines) are indicated on the right for each prey strain and *B. exovorus* offspring number. Source data are provided as a Source Data file. **g**, Representative time-lapse phase contrast microscopy images of *B. exovorus* growth using the *popZ*::Ω *C. crescentus* strains as a prey. The white arrowhead shows a rare case where 4 progenies are observed. Scale bars, 2 µm. Related to **Fig. 3**.

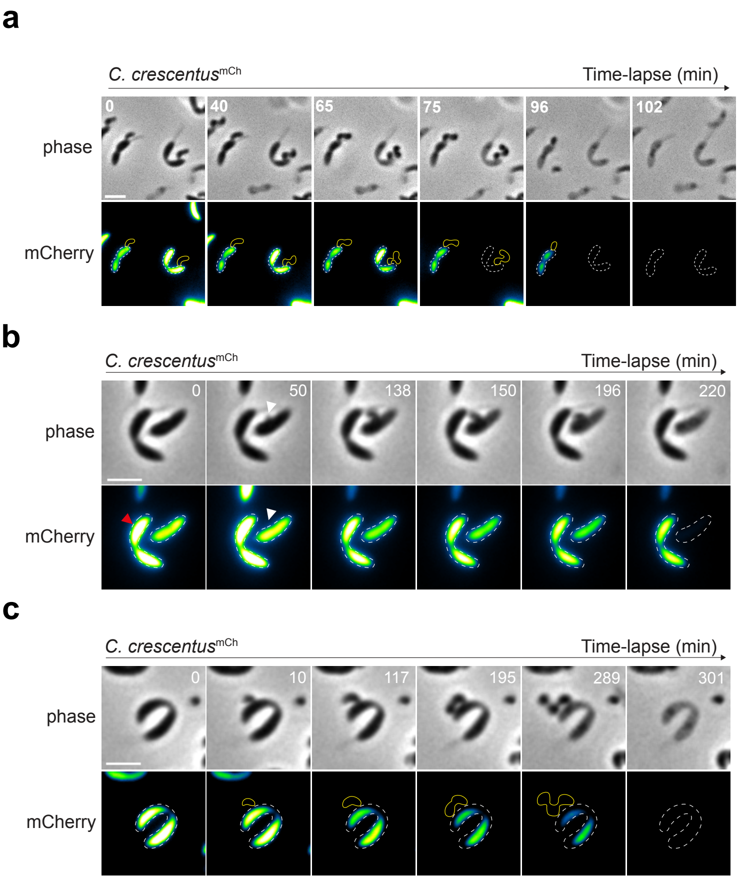

Supplementary Fig. 3 | *In situ* digestion of the prey's proteinaceous content by *B. exovorus*. The mCherry fluorescent signal is used as a reporter of the proteinaceous cytoplasmic content. **a-c,** Representative time-lapse microscopy images of the mCherry-producing *C. crescentus* (*C. crescentus*^{mCh}) predated by *B. exovorus*. *B. exovorus* cell outlines (yellow) and *C. crescentus* prey cell outlines (dashed white) were drawn manually based on the phase contrast images. The fluorescence signal was false colored with the GreenFireBlue colormap in Fiji to display changes in fluorescence intensity. **b,** White arrowhead highlights the predator binding site. Note the typical corkscrew-shaped elongation of the predator in the Z-axis. The red arrowhead shows an uninfected C. *crescentus*^{mCh} for which the mCherry fluorescent signal is not lost over time. Examples shown in **b** and **c** were selected from the same field of view. Scale bar, 2 µm. Related to **Fig. 4**.

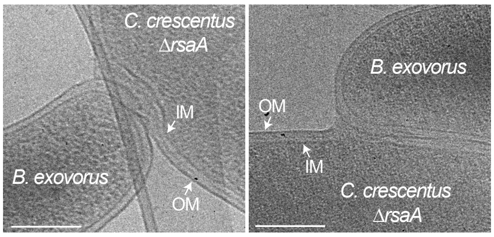

Supplementary Fig. 4 | Cryo-EM imaging of predator:prey junction using the Δ*rsaA C. crescentus* as a prey. Representative cryo-EM images of *B. exovorus* attached to the Δ*rsaA C. crescentus* cell surface. OM, outer membrane; IM, inner membrane. Scale bar, 0.2 μm. Related to **Fig. 5**.

SUPPLEMENTARY TABLE 1. Bacterial strains used in this study.

Bdellovibrio exovorus JSS		
**Strains**	**Description**	**Source**
**GL1867**	JSS	ATCC BAA-2330
Caulobacter crescentus		
**Strains**	**Description**	**Source**
**GL14**	Wild-type *C. crescentus* CB15N, Nal^R	Lab collection
**GL1866**	*C. crescentus* CB15N Δ*rsaA* (S-layer deficient mutant), Nal^R	Kind gift from Régis Hallez, UNamur
**GL2288**	CB15N cc1959::pHU1-yfp, Nal^R, $Gent^R$	Kind gift from Christine Jacob-Wagner, Stanford University
**GL2339**	CB15N *xylX*::pXbiofab-mCherry, Nal^R, Kan^R	This study
**Others**		
**GL1891**	*Agrobacterium tumefaciens*	Kind gift from Xavier De Bolle, UNamur
**GL1892**	*Sinorhizobium meliloti*	Kind gift from Xavier De Bolle, UNamur
**GL1893**	*Ochrobactrum anthropii*	Kind gift from Xavier De Bolle, UNAmur
**GL2073**	*Asticcacaulis excentricus*	Kind gift from Yves Brun, UDEM
**GL2076**	*Asticcacaulis biprosthecum*	Kind gift from Yves Brun, UDEM
**GL2078**	*Asticaccaulis benevestitus*	Kind gift from Yves Brun, UDEM
**GL2079**	*Brevundimonas subvibriodes*	Kind gift from Yves Brun, UDEM
**GL2080**	*Phenylobacterium lituiforme*	Kind gift from Yves Brun, UDEM
**GL2296**	*Hyphomonas neptunium*	Kind gift from Yves Brun, UDEM
