# Supplementary material for: Lifecycle of a predatory bacterium vampirizing its prey through the cell envelope and S-layer
Source: Nat Commun. 2024 Apr 27;15:3590. doi: 10.1038/s41467-024-48042-5 (PMC11055950; doi:10.1038/s41467-024-48042-5)
Supplement: Supplementary file 3 — Description of Additional Supplementary Files [file 41467_2024_48042_MOESM3_ESM.pdf]

Description of Additional Supplementary Files:

Supplementary Movie 1: Predator attachment onto the prey envelope occurs within seconds. Representative movie of *B. exovorus* predator cells attacking wild-type *C. crescentus* CB15N prey cells. Time is indicated in minutes:seconds. Related to Fig. 3.

Supplementary Movie 2: *B. exovorus* predator cells produce triplet progenies. Representative movie of *B. exovorus* predator cells growing onto wild-type *C. crescentus* CB15N prey cell bodies. Time is indicated in hours:minutes. Related to Fig. 3.

Supplementary Movie 3: The *C. crescentus* stalk can serve as a binding site for predator growth. Representative movie of a *B. exovorus* predator cell growing while attached to the stalk of its wild-type *C. crescentus* CB15N prey. Time is indicated in hours:minutes. Related to Fig. 3.
